# The Role of Dlc1 Isoform 2 in K-Ras2^G12D^ Induced Thymic Cancer

**DOI:** 10.1371/journal.pone.0040302

**Published:** 2012-07-05

**Authors:** Mohammad Golam Sabbir, Heather Prieditis, Esther Ravinsky, Michael R. A. Mowat

**Affiliations:** 1 Manitoba Institute of Cell Biology, CancerCare Manitoba, Winnipeg, Manitoba, Canada; 2 Department of Biochemistry and Medical Genetics, University of Manitoba, Winnipeg, Manitoba, Canada; 3 Department of Pathology, Health Sciences Centre, Winnipeg, Manitoba, Canada; University of Hong Kong, Hong Kong

## Abstract

The Deleted in liver cancer one (Dlc1) tumor suppressor gene encodes a RhoGTPase activating protein (RhoGAP). The Dlc1 gene has multiple transcriptional isoforms and we have previously established a mouse strain containing a gene trap (gt) insertion, which specifically reduces the expression of the 6.1 kb isoform (isoform 2). This gene trapped allele when homozygous results in embryonic lethality and the heterozygous gene trapped mice do not show an increased incidence of cancers, suggesting that cooperating oncogenic changes may be required for transformation. In the present work, we have studied the *in vivo* cooperation between oncogenic K-Ras2 and Dlc1 genes in tumourigenesis. We have observed an increase in invasive thymic cancers, including both thymomas and lymphomas, resulting in significantly shortened life spans in mice heterozygous for the gt Dlc1 allele and an inducible LSL-K-Ras2^G12D^ allele compared with the LSL-K-Ras2^G12D^ only mice. The heterozygous mice showed a high degree of metastasis in the lung. We have found tumour specific selective hypermethylation of the Dlc1 isoform 2 promoter and reduction of the corresponding protein expression in thymic lymphoma (TL) and thymic epithelial carcinoma (TEC) derived from the thymic tumours. The Dlc1 deficient thymic lymphoma cell lines exhibited increased trans-endothelial cell migration. TEC cell lines also exhibited increased stress fiber formation and Rho activity. Introduction of the three Dlc1 isoforms tagged with GFP into these cells resulted in different morphological changes. These results suggest that loss of expression of only isoform 2 may be sufficient for the development of thymic tumors and metastasis.

## Introduction

The Deleted in liver cancer 1 (Dlc1) tumour suppressor gene encodes a Rho GTPase activating protein (RhoGAP) that increases the intrinsic hydrolysis of GTP bound Rho to the inactive GDP bound form of Rho. The Dlc1 gene has been found frequently deleted or, down regulated by promoter hypermethylation in breast, lung, liver, colon and prostate tumours [1]–[6]. A recent study using representational oligonucleotide microarray analysis has shown heterozygous deletion of Dlc1 locus in ∼50% of liver, breast and lung tumours and 70% of colon cancers [7]. *In vitro* studies have shown that transfection of the Dlc1 gene can inhibit cell growth [5], [7], abolish *in vivo* tumour formation [2] and induce apoptosis. Recently, experiments using *ex vivo* knockdown of Dlc1 in p53 null and Myc induced liver progenitor cells have shown reduced survival after injection into mice [7].

The Dlc1 gene has at least three major transcriptional isoforms expressed under the influence of three alternative promoters in the mouse [8]. We have previously established a mouse strain containing a gene trap insertion, which specifically reduces the expression of the 6.1 kb transcriptional isoform (isoform 2) of Dlc1, thus creating a hypomorph [8]. Homozygous Dlc-1 gene trapped mice show an embryonic lethal phenotype [8], which phenocopies the Dlc1 exon 5 knockout mouse of Durkin et al. [6]. The heterozygous knockout and gene trapped mice are viable and do not show any increase in spontaneous tumours [6], [8]. This indicates that additional oncogenic events besides Dlc1 deletion are required for transformation.

It has been shown that in certain situations activation of the Ras signalling pathway arrests cell cycle inducing senescence rather than causing cell proliferation [9]–[12] through induction of p21^waf1^ [13], [14]. It has also been shown that in Ras activated cells, one requirement for Rho signalling is for the suppression of p21^waf1^ [15]. Again, in Ras transformed fibroblasts, the sustained ERK-MAP Kinase signalling favours the selection of high levels of active Rho-GTP to allow for down-regulating the high levels of p21^waf1^ [16]. Therefore, it is hypothesized that activation of the Rho pathway through loss of the Dlc1 RhoGAP expression will complement the Ras oncogene in cell transformation *in vivo*. It is also not known, which Dlc1 isoform is critical for tumour suppression. In this study, we report high incidence of metastatic thymic cancer in heterozygous Dlc1 isoform 2 gene trapped mice with an oncogenic K-Ras2^G12D^ allele. We also find selective hypermethylation of the Dlc1 isoform 2 promoter in these tumours.

## Results

### High Incidence of Thymic Cancer in the Dlc1^wt/gt^LSL-K-Ras2^wt/G12D^ Mice Infected with AdCMV-cre

**Figure 1:**
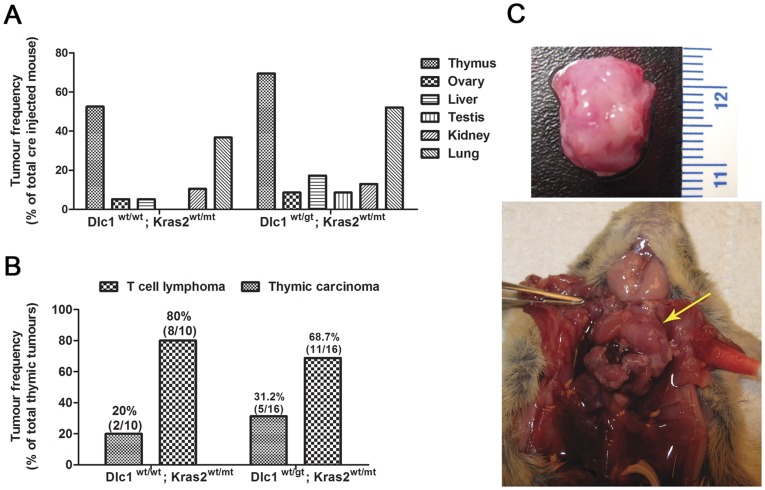
Tumour Frequency in Dlc1-K-Ras2^G12D^ mice after Cre-adenovirus tail vein injection. (A) Frequency of different primary tumour types. (B) Relative frequency of the different types of primary thymic cancers (C) Picture of enlarged thymus (arrow) in mouse and a dissected thymus with lymphoma.

**Figure 2:**
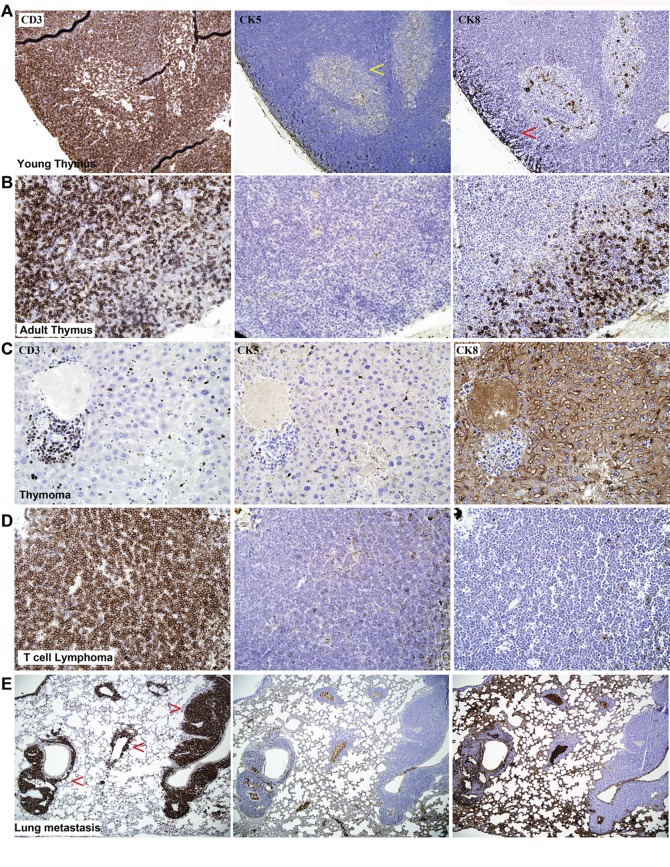
Immunohistochemistry of thymus and thymic tumours. Normal mouse thymus at 6 weeks (A) and 18 weeks (B) showing expression of T-cell marker (CD3) and different cytokeratin markers specific to thymic medullar epithelial cells (CK5) and cortical epithelial cells (CK8). Immunohistochemistry showing thymic epithelial carcinoma (C), T-cell lymphoma (D) and lung metastasis of T-cell lymphoma (F).

**Figure 3:**
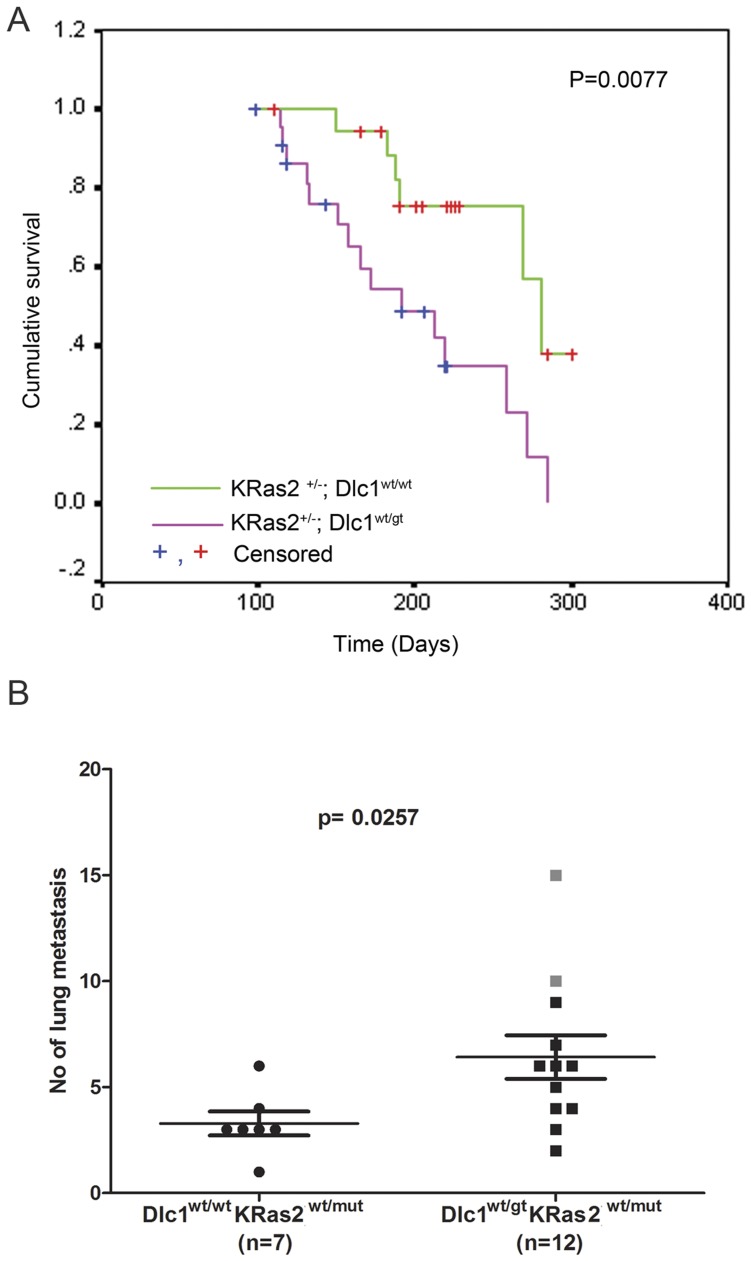
Kaplan–Meier survival probability curves and frequency of metastatic lung lesions in the LSL-K-Ras2^G12D^; Dlc1^wt/gt^ and LSL-K-Ras2^G12D^;Dlc1^wt/wt^ mice cohort. (A) Cumulative survival of heterozygous LSL-K-Ras2^G12D^;Dlc1^wt/gt^ and LSL-K-Ras2^G12D^;Dlc1^wt/wt^ mice following Cre expressing adenovirus injection via the tail vein. Survival time was defined as the time after injection to the death or culling, due to reaching humane end points. A total of 15 LSL-K-Ras2^G12D^; Dlc1^wt/wt^ and 18 LSL-K-Ras2^G12D^;Dlc1^wt/gt^ mice were used for the survival study. The censored data were mice that died of unknown causes with no detectable tumour or leukemia. (B) Frequency of metastatic lung tumours. The square shaped grey dots indicate the metastatic lesions derived from the mice that were the source of T Lymphoma cell lines 1 and 2. P value = 0.0257 (by the Mann-Whitney U test).

To test whether Dlc1 loss would cooperate with the K-Ras2 gene in transformation, we made use of the LSL-K-Ras2^G12D^ transgenic mouse [17]. To activate the oncogenic K-Ras2^G12D^ allele, Dlc1^wt/gt^;K-Ras2^wt/G12D^ (KD) and Dlc1^wt/wt^;K-Ras2^wt/G12D^ (K^+^) mice were injected with Cre-expressing AdCMV-Cre adenovirus via the tail vein. The majority of the mice developed primary thymic tumours within 6 to 9 months with 52.6% (10/19) of the K^+^ mice versus 69.5% (16/23) of the KD mice having tumours (Figure 1A & B, Table 1). The mice were usually culled for serious breathing trouble due to the compression of the chest cavity by the growing thymic tumours (Figure 1C). Upon necroscopy, some of these mice also showed neoplastic lesions in other organs including kidney, liver, lung, testis and ovary but, the majority of mice showed thymic tumours (Figure 1A, Table 1). The majority of the lung tumours were metastatic thymic lymphoma cells in origin (Figure 2E, Table 2). To determine overall survival, Kaplan–Meier analysis of the AdCMV-cre injected mice was carried out. This revealed significantly lower survival in the heterozygous KD gene trapped mice compared with K^+^ mice (P value = 0.0077, Figure 3A).

**Table 1:** Percent of Primary Tumour Types.

Genotype	Thymus	Ovary	Liver	Testis	Kidney	Lung
Dlc1 ^wt/wt^; Kras2^wt/mt^	52.6 (10/19)	5.3(1/19)	5.2 (1/19)	0	10.5 (2/23)	36.8 (7/19)
Dlc1^wt/gt^;Kras2^wt/mt^	69.5(16/23)	8.6(2/23)	17.3(4/23)	8.6(2/23)	13.04 (3/23)	52.1(12/23)

**Table 2:** Characterization of lung tumours.

Genotype	Lung metastasis	Tumour type	Number
Dlc1 ^wt/wt^;Kras2^wt/mt^	6(6/7)	T cell lymphoma	6
		Thymic carcinoma	0
Dlc1^wt/gt^;Kras2^wt/mt^	12(12/12)	T cell lymphoma	9
		Thymic carcinoma	2

To understand the nature of the thymic cancers, we performed immunohistochemistry using a T-cell specific marker CD3 and thymic epithelial cell specific markers, cytokeratin 5 (CK5) and cytokeratin 8 (CK8/Troma) [18]. In the normal young mice (6weeks old) the T lymphocytes were found evenly distributed in the both cortex and medulla regions of the thymus, whereas the medullar epithelial cells were strongly CK5 and cortical epithelial cells were strongly CK8 positive (Figure 2A). However, in the adult mice the T lymphocytes were predominantly shifted towards the central medulla region and the cortical epithelial cells were strongly CK8 positive with isolated patches of CK5 positive cells (Figure 2B). Using these markers we have found a significantly higher number of thymic lymphomas 80% (8/10) in the K^+^ mice versus 68.7% (11/16) in the KD mice (Figure 1B & 2D, Table 3). A smaller fraction (20% (2/10)) were predominantly thymic epithelial carcinomas (thymomas) in the K^+^ mice versus 31.2% (5/16) in the heterozygous KD mice (Figure 1B & 2C), as per the histological classification of Bernatz et al. [19]. The frequency of thymomas between the K+ and KD mice was not statistically significant. We have found only one tumour in a KD mouse, which showed a mixed thymoma type, which may have both T-cell lymphoma and thymoma.

**Table 3:** Characterization of thymic tumours.

Genotype	Thymic Tumours (%)	Tumour Type	Percent
Dlc1 ^wt/wt^; Kras2^wt/mt^	52.6 (10/19)	T cell Lymphoma (%)	80 (8/10)
		Thymic carcinoma (%)	20 (2/10)
Dlc1^wt/gt^;Kras2^wt/mt^	69.5(16/23)	T cell Lymphoma (%)	68.75 (11/16)
		Thymic carcinoma (%)	31.25 (5/16)

### Primary Thymic Tumours Frequently Metastasized to Lung

Immunohistochemistry also revealed that most of the thymic lymphoma tumours had metastasized to multiple foci in the lung. The majority of the lung metastases were of T-cell lymphoma in origin (Figure 2E, Table 2). Quantification of the lung metastatic lesions of each cohort shows significantly higher frequency of metastases in KD mice compared with the K^+^ mice (Figure 3B).

### Primary T-cell Lymphoma and Thymoma Cell Lines are Deficient in Isoform 2 Dlc1 Protein and Showed Increased RhoA Activity

**Figure 4:**
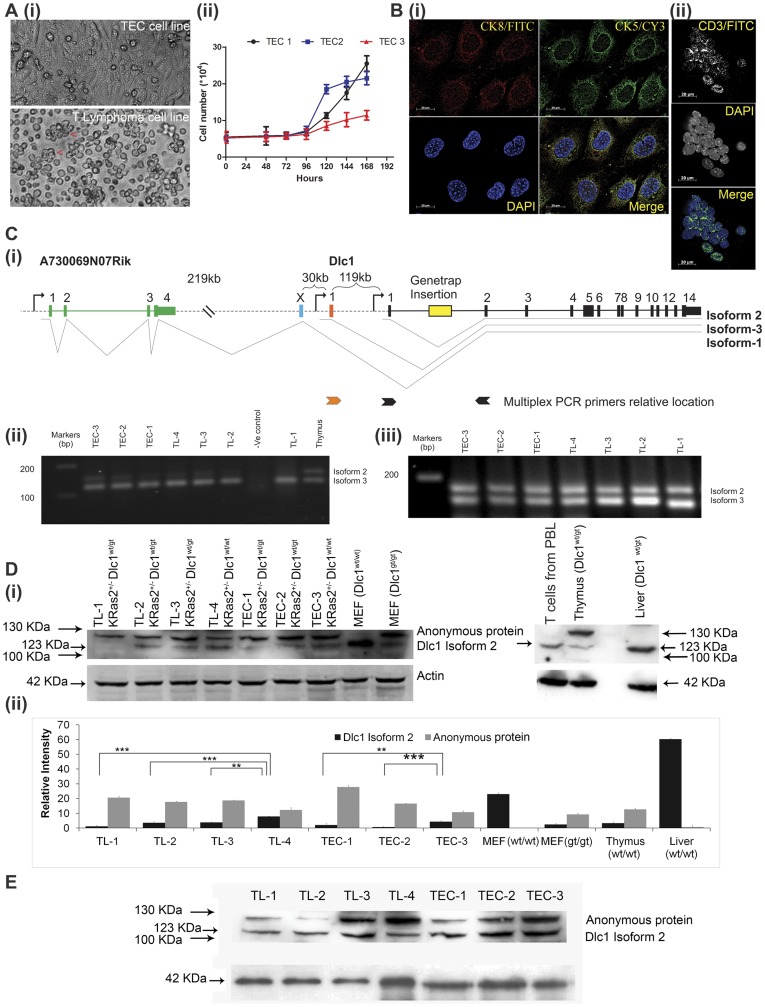
Thymic epithelial carcinoma (TEC) and Thymic lymphoma (TL) cells in culture and RNA and protein expression in TEC and TL cell lines. A(i) Bright field micrograph of the TEC and TL cells in culture.(ii) Proliferation rate of TEC cell lines. The cells were plated onto 6 well plates and then the cell growth was analyzed by counting cell numbers consequently for 7 days. B(i) Immunofluorescence showing Thymic epithelial cells expressing of CK8 and CK5 and (ii) T cell lymphoma showing expression of CD3. (**C**) **Dlc1 mRNA expression in TEC and TL cell lines.** (i) Diagrammatic representation of the Dlc1 locus and splicing pattern of the three major isoforms. The strategy and primers used for relative quantification of the Dlc1 isoforms 2 and 3 using multiplex RT-PCR. (ii) Multiplex RT-PCR showing relative intensity of isoform 2 and 3 mRNA expression in T-cell lymphoma and thymoma cell lines.(iii) Multiplex PCR showing relative intensity of isoform 2 and 3 mRNA expression in 5-AzaC treated TL and TEC cell lines (**D**) **Dlc1 protein expression:** (i) Dlc1 protein from T-cell lymphoma and thymic epithelial carcinoma cell lines as well as normal adult thymus and liver tissue from C57Bl/6J mouse and T cells from peripheral blood lymphocytes (PBL). (ii) Plot showing the relative intensity of Dlc1 123 KDa (isoform 2) and 127 KDa anonymous proteins in T-cell lymphoma and carcinoma cell lines as well as adult thymus and liver tissue of C57Bl/6J mouse (*p<0.05, **P<0.01, ***P<0.001, ****P<.0001). E. Dlc1 protein expression from 5 AzaC treated TL and TEC cell lines.

We established 4 thymic lymphoma (TL) and 3 thymic epithelial carcinoma (TEC) cell lines (Figure 4A) from the primary tumours to study the expression of Dlc1 protein. These tumours were either CD3+ T-cells or CK 5 or CK 8 positive epithelial cells (Figure 2C &D). The TL and TEC cell lines spontaneously immortalized and were grown up to 20 passages without any decrease in growth potential. Cell proliferation rates, by a cell counting method, showed that the TEC 1–2 (KD) cell lines had an increased rate of growth in comparison with the TEC 3 (K+) cell line(Figure 4A/ii). The TL cell lines showed no significant difference in the rate of cell proliferation in culture.

In our previous study, we have reported the existence of two different molecular weight isoforms of Dlc1 protein in different mouse tissues and in mouse embryonic fibroblasts (MEF), which presumably originates from the isoforms 2 and 3 mRNAs or by post-translational modifications [8]. The regular 123 KDa Dlc1 protein (isoform 2) is expressed at a low level in the adult thymus. Whereas, the 127 KDa cross reacting protein is expressed at a higher level in the thymus when compared with the liver in western blots (Figure 4D/i). Interestingly, the thymoma and lymphoma cell lines showed lower expression of the 123 KDa protein band and the corresponding isoform 2 mRNA when compared with normal thymus (Figure 4C & D/i–ii). The TL4 and TEC3 cell lines from K+ mice showed levels Dlc1 expression comparable with wild type thymus. (Fig. 4C/i–ii). The TL1, 2 & 3 and TEC 1& 2 lines showed lower levels compared with the lines from K+ mice (Figure 4D). Also, there was a significant increase in the 127 KDa protein band in the tumours compared with the thymus (Figure 4D). In our previous study as well as in the present study, we have noticed that the protein expression level of 127 KDa protein increases in presence of reduced levels of the 123 KDa protein, which is particularly evident in the homozygous gene trapped mouse embryonic fibroblast cells (Figure 4D). Most of the lymphoma and TEC cells also showed increased expression of the 127 KDa protein (Figure 4D).

To determine if the additional high molecular bands are phosphorylated forms of the Dlc1 protein, the cellular lysates were treated with alkaline phosphatase. The continued presence of the 127 KDa band after phosphatase treatment indicates that it is not a phosphorylated form of regular 123 KDa Dlc1 protein (Figure 4C/i & E).

To identify the 127 KDa cross reacting protein, immunoprecipitates using the Dlc1 antibodies were subjected to mass spectrometry. We have been able to identify 7 peptides corresponding to the consensus region of Dlc1 common to all isoforms and therefore, the mass spectrometry was inconclusive about the existence of the other Dlc1 isoforms (supplementary Figure S1).

**Figure 5:**
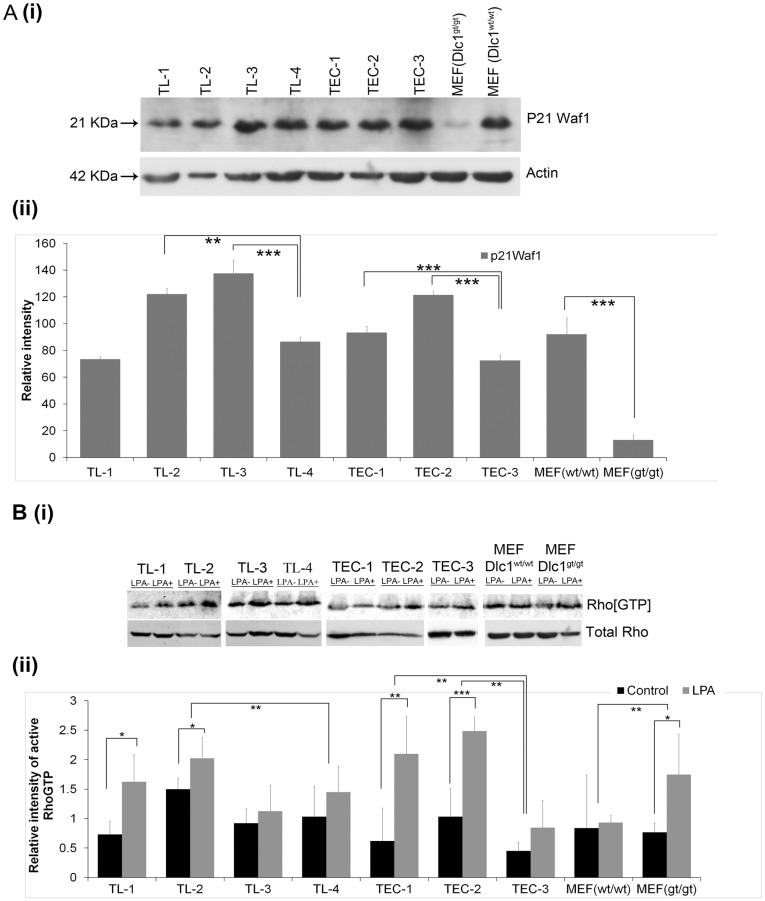
Western blots showing p21^waf1^ expression and active Rho in TEC and TL cell lines. A(i) p21^waf1^ protein in T-cell lymphoma and carcinoma cell lines, (ii) Plot showing the relative intensity of p21^waf1^ protein in T-cell lymphoma and carcinoma cell lines relative to actin. Means and standard error of mean were determined from at least five independent experiments. (*p<0.05, **P<0.01, ***P<0.001, ****P<.0001). (B) **Measurement of active RhoGTP in cell lines**. (i) RhoGTP pull down assay showing constitutive and LPA induced levels of active RhoGTP in T-cell lymphoma and thymic epithelial carcinoma cell lines. (ii) Plot showing the RhoGTP to total Rho protein ratio by scanning the relative intensity of the protein bands in non-treated and LPA induced in TEC, TL, MEF cell lines. Means and standard error of mean determined from four independent experiments. (*p<0.05, **P<0.01, ***P<0.001 and ****P<.0001 by Student t test and two way ANOVA test.

The majority of the lymphoma and TEC cell lines also showed high expression of the p21^waf1^ protein in contrast to homozygous gene trapped mouse embryo fibroblast cells where p21^waf1^ expression was decreased (Figure 5A/i–ii). The expression level of p21 protein was comparable in MEF^+/+^ and the TL4 and TEC3 cell lines, which have wild type Dlc1 alleles However, the heterozygous genetrap Dlc1 allele containing TL 2/3 cell lines and the TEC1/2 cell lines showed significantly higher levels of p21 protein expression when compared to their corresponding wild type, TL4 and TEC-3 cell lines respectively. In contrast, the heterozygous TL1 line, with the lowest Dlc1 expression, showed p21 expression comparable to wild type MEF^+/+^ and TEC 4 cells. Therefore p21 expression does not correlate with Dlc1 expression in thymic cell lines, unlike in fibroblasts.

All the thymoma and lymphoma cell lines showed varying degrees of activated RhoA when treated with lysophosphatidic acid (LPA) (Figure 5B/i–ii). The LPA induction of active RhoA was significantly higher in two lymphoma (TL-1&-2) and two TEC lines (TEC-1&-2),which also showed the lowest Dlc 1 isoform 2 levels, when compared with the corresponding wild type cell lines (Figure 5B/i–ii). This was similar to homozygous Dlc1^gt/gt^ cells, indicating that these lines may have inactivated the wild type Dlc1 allele.

### Tumours have Activated K-Ras2 G12D Mutation

**Figure 6:**
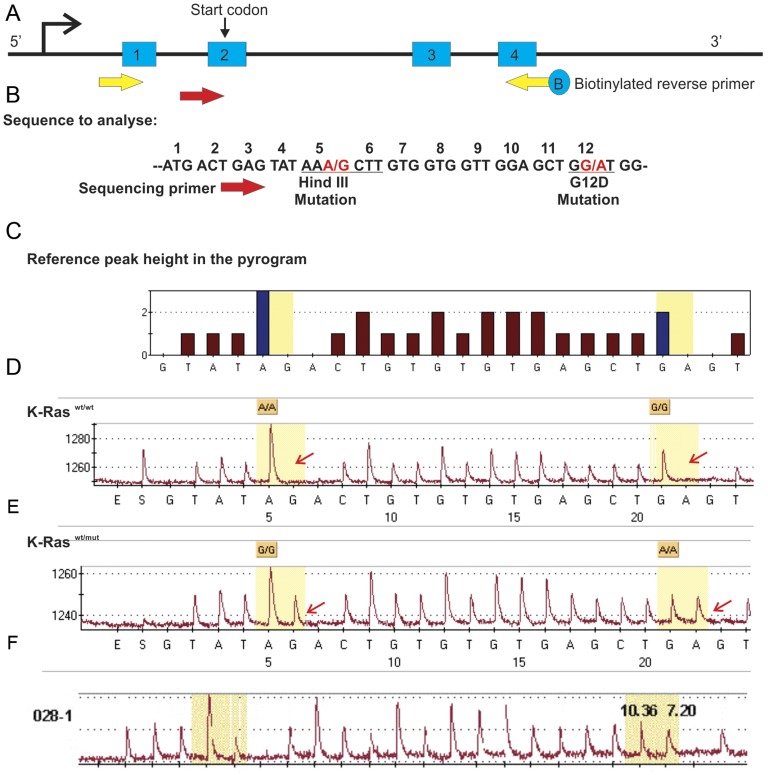
K-Ras2^G12D^ mutation analysis. (A) Diagrammatic representation of The PCR strategy for amplifying K-Ras2 cDNA. (B) K-Ras2 sequence showing location of the mutations and the pyrosequencing primers relative positions. (C) The program’s reference peaks showing the dispensation order and sequence. (D) A pyrogram of wild type K-Ras2 allele showing no mutation, (E) one mutant allele in a tumour cell line showing the presence of mutant as well as wild type alleles and (F) the mutant allele in a micro dissected primary tumour. In Figure E, the difference in relative intensity between the normal and mutant allele specific nucleotide base indicates presence of normal cells in the tumour.

To determine that the tumours were due to the activation of K-Ras2^G12D^ allele, we extracted cellular RNA from the primary tumours and the tumour cell lines and then performed pyrosequencing based mutation analysis for K-Ras2^G12D^ mutation (Figure 6). All the primary tumours showed expression of the mutant K-Ras2 allele (Figure 6F). However, the expression of the mutant allele was less than 50% in the primary tumours as revealed by the pyrogram, presumably due to normal cell infiltration (Figure 6D & E). In the tumour cell lines the mutant and wild type allele were expressed at equal levels indicating absence of normal cell contamination (Figure 6F).

### Dlc1 Isoform 2 Promoter Methylation in Thymomas and Lymphomas

**Figure 7:**
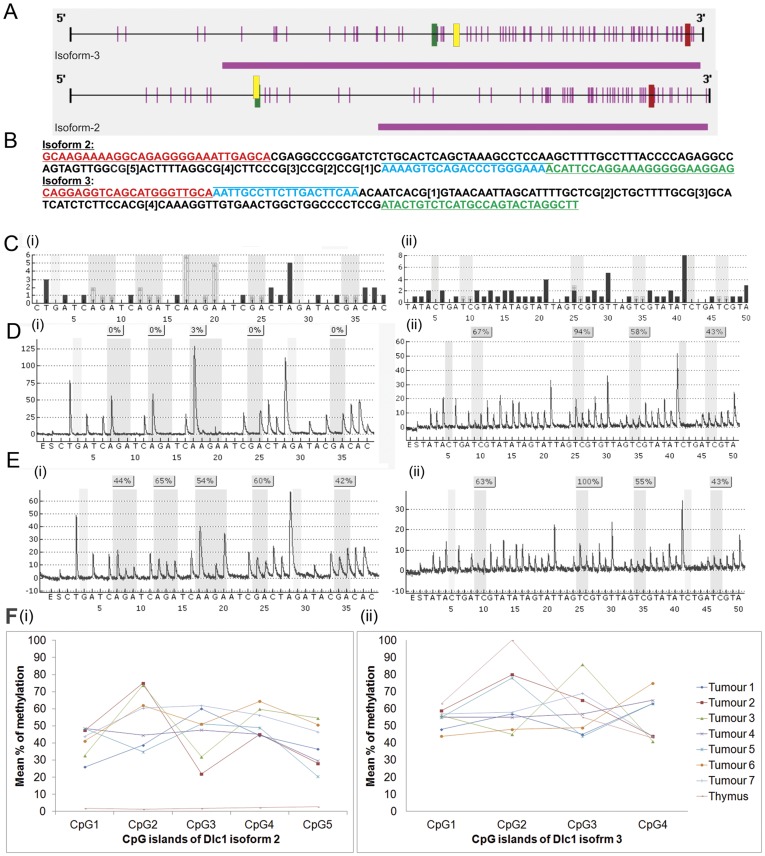
Methylation analysis of the CpG islands in Dlc1 promoter. (A) Diagrammatic representation of the CpG islands (pink horizontal bar), Sp1 promoter binding site (yellow vertical bar), translation start codon (red vertical bar close to 3′end), and the CpG islands selected for methylation analysis (dark green vertical bar) in the promoter region of Dlc1 isoforms 2 and 3. (B) DNA sequence and location of the sequencing primers for pyrosequencing (underlined red and green - forward and reverse PCR primer respectively, italics-sequencing primer). (C) Dispensation order and reference pyrogram of isoforms 2(i) and (ii). Pyrogram of the normal (D) and tumour tissue (E) for isoforms 2 (i) and 3(ii). F: Plot showing the mean percentage of methylation in the different CpG sites on the Dlc1 isoforms 2 and 3 promoters in tumours and normal thymus.

In order to understand the mechanism for reduced Dlc1 protein expression in tumour cell lines, we have studied the methylation status of Dlc1 isoform 2 and 3 promoters by bisulfite treatment of DNA followed by PCR and pyrosequencing (Figure 7). We have identified strong CpG islands in the promoters of Dlc1 isoforms 2 and 3 using the Methyl Primer Express Software version v1.0 (Applied Biosystems) (Figure 7A). Further analysis of these CpG islands using Matinspector (Genomatix software suite) revealed two suitable sites for bisulfite sequencing close to a Sp1 promoter binding site on Dlc1 isoform 2 and 3 promoters. We have targeted five CpG islands located 502 bp upstream of the start codon for the isoform 2 and four CpG islands located 851 bp upstream of the start codon of isoform 3 (Figure 7B). DNA from microdissected primary tumours as well as tumour cell line derived DNA revealed extensive methylation of the Dlc1 isoform 2 promoter (30–70%) in all 5 CpG islands compared with normal thymus DNA (Figure 7F). The promoter of the Dlc1 isoform 3 has been found to be highly methylated (40–100%) in normal tissue and in primary tumours for all the 4 CpG islands analysed (Figure 7F). The frequency of methylation in the primary tumour also matched with the methylation pattern observed in the cell line DNA.

**Figure 8:**
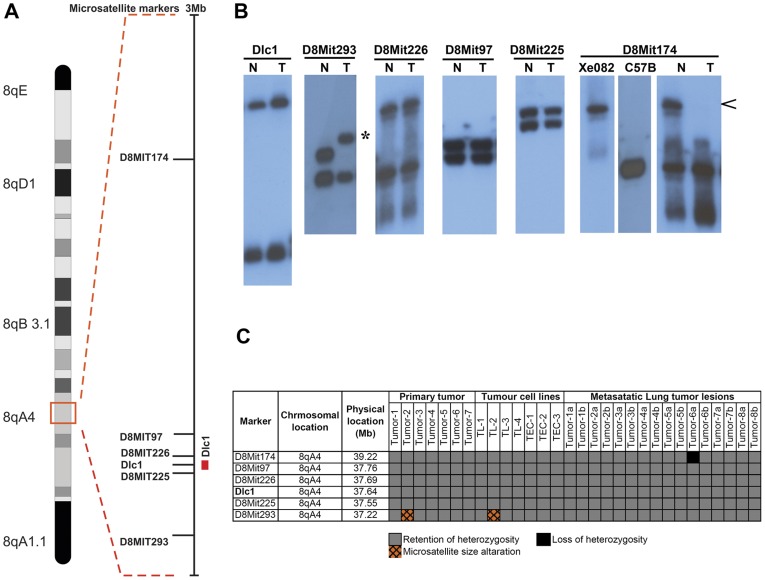
Deletion mapping of Dlc1 locus. (A) Diagrammatic representation of the relative position of the different microsattelite markers used for loss of heterozygosity analysis. (B) Representative autoradiographs showing the alleles specific to the different microsatellite markers. Arrow indicates LOH of one allele and the asterisk indicates microsatellite size alteration (MA) of one allele, N-Normal, T-Tumour (C) Allele status of the chromosome 8qA4 markers in the thymic tumours and TEC and TL cell lines as well as corresponding primary and metastatic tumours; RH - Retention of homozygosity. Tumours 1–7 are primary thymic tumours corresponding to TL and TEC cell lines. The subcategory a and b under Tumour 1–7 represents DNA obtained from two independent lung metastatic lesions of the corresponding primary tumour respectively.

Deletion of Dlc1 locus has been reported to be responsible for loss of protein expression in different human tumours. Therefore, to examine the allelic loss at the Dlc1 locus, we used polymorphic microsatellite based loss of heterozygosity (LOH) analysis around the Dlc1 gene locus (Figure 8). The LOH analysis revealed no significant loss at the Dlc1 locus in the majority of tumours; however, we have found microsatellite size alteration for the D8Mit293 marker in one tumour as well as the corresponding cell line DNA (Figure 8C). We have also found heterozygous loss of D8Mit174 marker in a metastatic lung lesion (Figure 8C).

In order to find out whether methylation of the Dlc1 promoter was responsible for decreased expression of Dlc1 mRNA and protein, we treated the tumour derived cell lines with DNA demethylating agent 5′-azacytidine (5 AzaC) and then studied the expression of Dlc1 mRNA and protein. Interestingly, treatment with 5 AzaC increased the expression of Dlc1 isoform 2 as compared to the control tumour cell lines (Figure-4C/iii & E). The multiplex RT- PCR using cDNA derived from 5 AzaC treated cell lines also showed uniformly high expression of Dlc1 isoform 2 and 3 mRNA in all the treated cell lines.

### Altered Cytoskeleton Structure and Increased Cellular Motility in Tumour Cells

**Figure 9:**
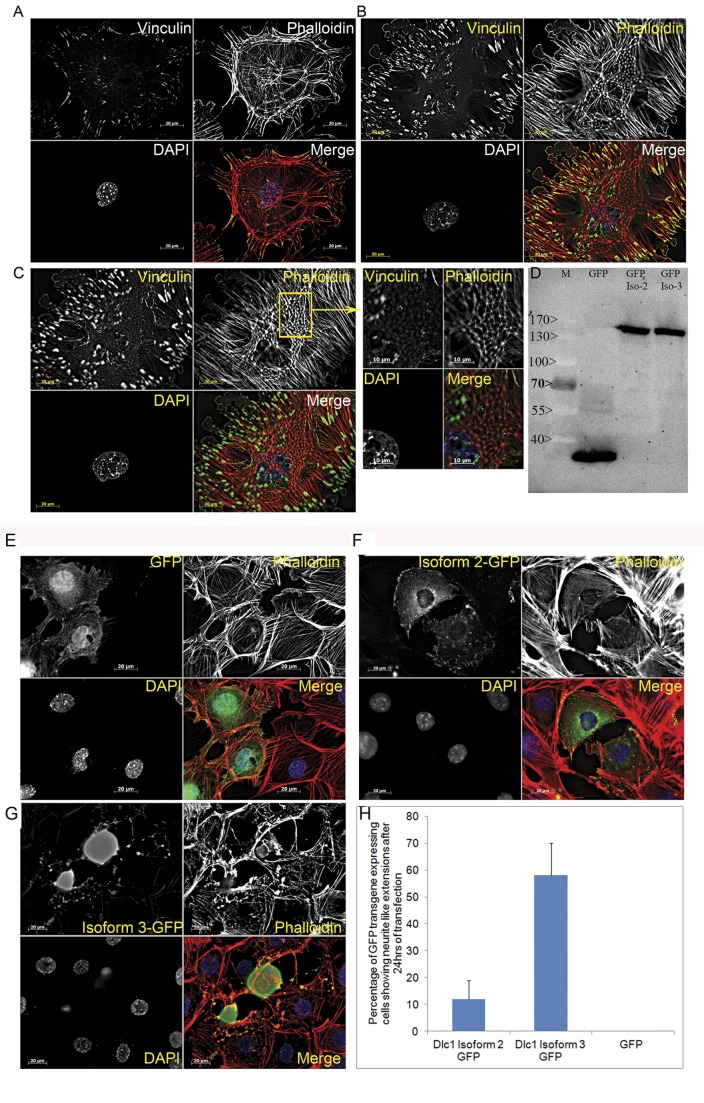
Comparison of stress fibre formation in thymic epithelial and TEC cells and effect of GFP tagged Dlc1 isoform 2 and 3 expression on cell morphology and stress fibre formation. (A) Normal thymic epithelial cell showing stress fibre formation in the form of an annular transverse arc. (B) Thymic epithelial carcinoma cell (Z stack-1) showing extensive ventral stress fibre formation and increasing frequency of focal adhesions, (C) the same tumour cell showing a different structural organization of vinculin and actin at Z stack -7 on the dorsal surface of the cell. (D) Western blot showing transient expression of Dlc1 isoform 2 & 3 proteins tagged with C terminal GFP in the TEC cell line. Thymic epithelial carcinoma cells transfected with (E) pEGFP C1 vector, (F) Dlc1 isoform 2 GFP construct; (G) Dlc1 isoform 3 GFP construct. (H) Bar diagram representing the relative number of cells that are showing neurite like extensions expressed as a percentage of total GFP transgene expressing cells.

Since increased Rho activity is associated with stress fibre formation and cellular motility, we examined the frequency of stress fibre formation and focal adhesion in the cultured TEC cell lines and compared with normal thymic epithelial cells. The tumour cells contained significantly higher number of stress fibres (26±11/1000 µm^2^) and focal adhesions (28±18/1000 µm^2^) in comparison with normal cells with 8±9/1000 µm^2^ and 7±15/1000 µm^2^ frequency respectively (P value <0.0001) (Figure 9). The average size of the tumour cells (8–12×10^3^ µm^2^) was also significantly larger than their normal counterpart (6–8×10^3^ µm^2^) (p value = 0.016). Moreover, the tumour cells showed more ventral stress fibres [20] whereas, in normal thymic epithelial cells stress fibres were mostly organised as transverse arcs [21] (Figure 9 A–C). We have also observed stress fibres organised in triangular mesh like networks overarching the nucleus at the dorsal cell surface in the thymoma cells (Figure 9C).

The stress fibres were abolished when TEC cells were transfected with GFP tagged full length Dlc1 isoform 2 (Figure 9E–G). The expression of the GFP-Dlc1 fusion proteins were also verified in a western blot using anti GFP antibody (Figure 9D). The GFP-Dlc1 isoform 2 fusion protein was found localized to the focal adhesions (Figure 9F). We have also noticed that the tumour cells showed profuse formation of “neurite like” cytoplasmic extension or filipodial structures along with destruction of stress fibre assembly when transfected with GFP tagged Dlc1 isoform 3 (Figure 9H). All Dlc1 transfected cells eventually died 3–4 days after transfection.

**Figure 10:**
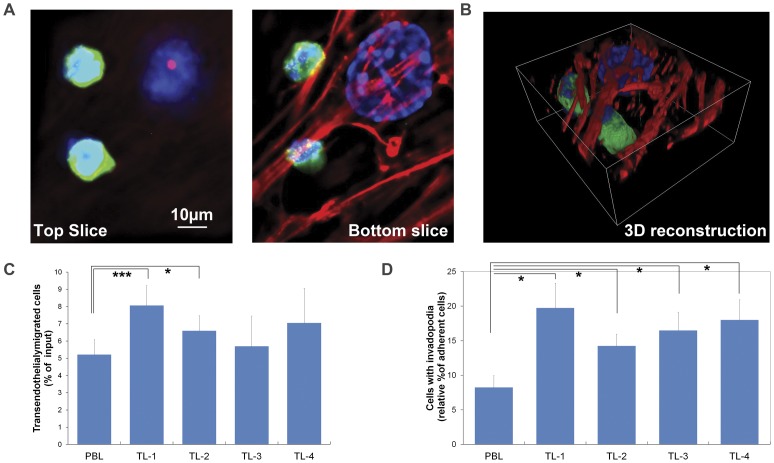
Transendothelial migration and invadopodia assay of the T lymphoma cells. (A) Depicted are two TL cells that had invaded between the endothelial cells. The TL cells were stained with T-cell receptor marker specific anti-CD3 antibodies and the endothelial cells were stained with DAPI and TRITC-Phalloidin. The top panel shows representative Z stack images of the top slice (above the endothelial cells) and the arrow indicates pseudopod formation (B) Transendothelial migration assay of T lymphoma cells. Three dimensional reconstructions showing two TL cells that had invaded the confluent endothelial monolayer. Z stacked images taken at 0.5 µm intervals have been used for reconstruction of the 3D image. (C) The percentage of trans-endothelial migrated T-lymphoma cell lines. The mean and the standard error are shown for 5 experiments (D) Percentage of cells that showed a leading edge pseudopod structure beneath the bEND.3 cells. The mean and the standard error of mean are shown for 4 experiments in which more than 200 cells were analyzed per experiment. (*p<0.05, **P<0.01, ***P<0.001 and ****P<.0001 by Student t test).

To determine if the T-lymphoma cells with lower Dlc1 levels showed increased extravasation and migration, a transendothalial migration assay was carried out (Figure 10). The invasion rate of the T lymphoma cells were found significantly higher in lymphoma cell line 1 and 2 in comparison with normal peripheral blood (PBL) derived T-cell line (p value = 0.0007 and 0.021 respectively, Figure 10C). However, the remaining 2 lymphoma cell lines, with comparatively higher Dlc1 expression, did not show significant differences in invasion (p value = 0.068). All the lymphoma cell lines also showed significantly higher rate of leading edge pseudopodal structure when compared to the normal PBL T-cells (P value <0.04) (Figure 10D). The increased leading edge pseudopod formation and transendothelial migration in lymphoma cell line 1 and 2 matched with increased metastasis in the corresponding mice cohort (Figure 3).

## Discussion

The Dlc1 gene like other tumour suppressor genes, for example TP53, APC and BRCA1, uses alternative promoters to yield at least three full length isoforms in mice [22]–[25]. There is growing evidence of selective use of alternative promoters, which regulates differential transcription, may be involved in initiation and progression of cancer [26]. The aberrant use of one promoter over another in genes, such as TGFB3, LEF1 and CYP19A1, is directly linked to cancerous cell growth (reviewed by [26]). The alternative promoters used by the Dlc1 gene are evolutionarily conserved and common among the other Dlc family members [27], [28]. The Dlc1 gene has three major alternative transcripts which were previously described as 7.4, 6.1 and 6.2 Kb transcript by Sabbir et al. [8]. These isoforms have been designated as Isoform 1, 2 and 3 respectively in the UCSC Genome Browser on Mouse July 2007 (NCBI37/mm9) Assembly. For this discussion, we will use the UCSC nomenclature of the isoforms. Previously, it has been shown that all three transcriptional isoforms of Dlc1 gene are expressed to varying degrees in different mouse tissues but, their individual role in tumourigenesis is still unknown [8]. The spatio-temporal expression of the human Dlc1 isoforms equivalent to mouse Dlc1 is still lacking. Recently, Low et al. have identified a novel Dlc1 isoform 4 in humans, which corresponds to the Dlc1 isoform 3 in the mouse and it has been found silenced in multiple carcinoma lines by promoter hypermethylation [29]. The genetrap mouse that we have used in our study has previously been shown to trap Dlc1 isoform 2 and reduces its expression but, not isoforms 1 and 3 [8]. The isoform 3 promoter showed a high degree of constitutive methylation in normal thymus tissue and thymic tumour cells. Both these cell types still showed expression of isoform 3 mRNA. High methylation of Dlc1 promoter with continued expression of the gene has also been reported for isoform 2 in canine lymphoma [30]. These results indicate that methylation status alone may not be sufficient to predict expression of Dlc1 isoform 3, at least in the thymus and T lymphocytes. These results also suggest that selected loss of Dlc1 isoform 2 expression may be sufficient for tumor progression.

The Dlc1 genetrap alone is unable to induce tumours however, in presence of K-Ras2^G12D^ mutation, it increases tumour and metastasis frequencies compared with K-Ras2^G12D^ mutation only mice, indicating an additive effect. The conditional K-Ras2^G12D^ mouse used in this study was previously reported to develop adenocarcinoma in the lung upon intranasal delivery of AdenoCre virus [17]. Another transgenic mouse model, LA1-K-Ras2, where the K-Ras2^G12D^ allele can be activated by a somatic intrachromosomal recombination event, showed high incidence of lung tumours but, also a 30% incidence of thymic lymphoma and other tumour types [31]. In our study, we have found predominantly thymic tumours along with neoplastic lesions in many other organs using tail vein injection of AdenoCre. Previous studies have shown that tail vein injection of AdenoCre mostly targets the liver, spleen and kidneys and to a lesser extend the thymus, heart and lung tissue [32], [33].

The overall frequency of neoplastic lesions and thymic tumours was significantly higher in KD mice suggesting that trapping Dlc1 isoform 2 may have provided an advantage to the initiation of tumourigenesis. The KD mice also showed a significant reduction in overall survival. The increase in lymphoma metastases in the lungs of the KD mice may be the cause of reduced survival in these mice. The majority of the lung tumours in our study were thymic lymphoma metastases. The difference in the initiating organ for tumourigensis between Jackson et al. [17] and our study is mainly due to the mode of AdenoCre delivery. It may also be due to the fact that the mouse thymus normally expresses a lower level of the Dlc1 isoform 2 protein. It is possible that the Dlc1 isoform 2 deficient environment in the thymus may have given cells expressing the oncogenic K-Ras2^G12D^ allele a selective advantage. As well, there was a comparable reduction in the Dlc1 isoform 2 protein in the KD cell lines in comparison with K+ only cell lines, which also coincided with an increased rate of tumourigenesis.

Interestingly, expression of an anonymous 127 KDa protein increased significantly in all tumour cell lines that had reduced levels of isoform 2 protein. The mutual exclusiveness was more evident in the thymoma cell lines. Evidence for differences in function between the Dlc1 isoforms comes from the transfection experiments into the thymoma cell line. Cells expressing the Dlc1 isoform 2-GFP construct showed a reduction in stress fibre formation and spreading of the cells. In contrast, Dlc1 isoform 3-GFP expressing cells showed cell rounding and production of “neurite-like” extensions. A similar phenotype was previously seen when an amino terminal deleted form of Dlc1 or just the RhoGap domain were transfected into breast cancer cells [34]. This suggests that the novel exon 1 of isoform 3 may change either localization or the RhoGap activity of Dlc1. It was previously suggested that the Dlc1 sterile alpha motif (SAM) domain acts as a negative regulator of the Dlc1 RhoGAP catalytic activity [34]. Since the Dlc1 isoform 3 SAM domain is still intact, this suggests that the subcellular localization may be critical for production of the “neurite- like” extensions and not removal of a negative regulatory sequence. The usual mechanisms for down regulation of Dlc1 protein in tumours is either deletion of the Dlc1 locus or hypermethylation of the Dlc1 promoter [28]. Somatic mutations are rare for the coding region of Dlc1 gene in different tumours, reviewed by [28] and so far there is no report on the association of existing single nucleotide polymorphisms in Dlc1 with the outcome of the disease [35]. In our study, we have found a significantly higher percentage of tumour specific methylation of the Dlc1 isoform 2 promoter, which correlates with the reduced Dlc1 isoform 2 protein expression in these tumours. The mean percentage of methylation found in our study is comparable with the methylation frequency in different human tumours [28]. The methylation pattern of the isoform 3 promoter did not show any change and remained highly methylated in the adult thymus as well as in the tumours. This indicates complex regulatory mechanism controlling selective methylation of alternative promoters. Recently, it has been shown that in Myc induced T-cell lymphoma, there is a selection for specific rare DNA methylation events during the course of tumour development *in vivo* [36]. Thus, our results indicate that in the K-Ras2 induced thymic tumours, selective methylation of the promoter of Dlc1 isoform 2 may lead to a malignant phenotype in thymus.

The Dlc1 gene has recently been viewed as a metastasis suppressor gene [28]. The reduced expression of Dlc1 mRNA has been correlated with the invasiveness of hepatocellular carcinoma (HCC) [37]. Re-expression of Dlc1 inhibited the mobility and invasiveness of HCC, breast, ovarian and lung cancer cell lines. In our study, we have found significantly higher metastases of the thymic cancers to the lung. The increased metastasis found in the KD mice coincides with higher levels of active RhoA activity. Since T lymphocytes are intrinsically mobile and migratory, what selective advantage would active Rho have on T-cell lymphoma metastasis? Using transformed T cell lines, Gomez et al. have shown that active Rho prevents apoptosis after interleukin withdrawal, through induction of Bcl2 [38]. Costello et al. have shown that Rho controls a p53 dependent survival checkpoint in pre-T cells and that Rho is also important for p53-independent survival of CD4/8 double positive cells [39]. Rho activation can induce integrin-mediated adhesion in mouse thymocytes [40]. Cell-cell or cell-extra cellular matrix adhesion through integrin- receptors is necessary for T cell migration and maturation [41]. Changes to the actin myosin cytoskeleton can also change T-cell migration rates [42], [43]. Alteration in anyone of these Rho mediated functions could increase the metastatic potential of T lymphoma cells.

Initially, we hypothesized that the reduced Dlc1 expression would complement the K-Ras2 oncogene in tumourigenesis through RhoA’s suppression of p21^waf1^ protein expression. This has been found true in the case of MEF cells, where reduction of Dlc1 protein expression significantly decreased p21waf1 expression in the MEF Dlc1^gt/gt^ cell line. p21 deficiency contributes to the development of a wide variety of tumour types in mice, which include skin, colon, intestine, pituitary, thyroid, mammary gland, salivary gland, connective tissue and histiocytic sarcomas [44]–[52]. On the contrary, in our study we have found increased expression of p21^waf1^ in the heterozygous Dlc1 genetrap allele containing T-cell lymphoma and thymoma cell lines. These cell lines have considerably lower level of Dlc1 protein when compared with the corresponding wild type Dlc1 allele containing T cell lymphoma and thymoma cell lines. This may indicate to the fact that RhoA mediated suppression of p21 expression thorough loss of Dlc1 may be cell type specific.

In brief, our finding indicates that Dlc1 isoform 2 undergoes selective hypermethylation in oncogenic K-Ras2 induced thymic tumours and significantly decrease the overall survival in mice. These results indicate that at least in thymic tumours the loss of only Dlc1 isoform 2 expression is sufficient for tumour progression.

## Materials and Methods

### Transgenic Mice

The Dlc1 gene trap mice were generated in our lab and described previously [8]. The K-Ras2^tm4Tyj^ mice were purchased from National Cancer Institute Mouse Consortium (Frederick, MD, USA). All experiments were performed in accordance with the Canadian Council on Animal Care (CCAC) and were approved by the University of Manitoba Animal Protocol Management and Review Committee before experimentation.

### Cell Culture

Thymic tumours were gently removed without squeezing the tissue. The tumours were cut into approximately 1–2 mm fragments and trypsinized and cultured in serum free media [8] as well as in RPMI-1640 complete medium supplemented with 10% v/v fetal calf serum (FCS) Invitrogen (Burlington, ON, Canada), 2 mM L-Glutamine, and 55 µM β-mercaptoethanol, and antibiotics. The tumour cells spontaneously immortalized and sub-cultured up to 20 passages. Most of the gene expression studies have been done with cultured cells at 16–20 passages.

### Vector Constructs and Viral Infection in Vivo

The Adenovirus vector expressing Cre, under control of the CMV promoter, used in this study was obtained from Vector Biolabs, (Philadelphia, PA, USA). The Dlc^gt/wt^ mice were crossed with K-Ras2^G12D/wt^ mouse to get a Dlc^gt/wt^; K-Ras2^G12D/wt^ mouse. Eight week Dlc^gt/wt^; K-Ras2^G12D/wt^ and the K-Ras2^G12D/wt^ mice were injected with 1×10^8^ plaque forming units of AdCre virus/0.2 ml phosphate buffered saline (PBS) via the tail vein. The survival data were analysed using a log-rank test of the Kaplan-Meier estimate of survival.

### Fluorescence Microscopy

Cells for direct/indirect immunofluorescence were plated into 6-well plates pre-coated with 0.1% gelatin and 10 mg/ml fibronectin in Alpha MEM at a density of 2×10^5^ cells per well. The cells were transfected with GFP tagged Dlc1 isoforms using Lipofectamine (Invitrogen) at 70% confluency and then grown overnight. The next day cells were trypsinized and then grown O/N on coverslips coated with on 0.1% gelatin. Cells were fixed with 4% paraformaldehyde and permeabilized with 0.2% Triton X-100 and blocked with 1% skim milk powder/0.5% Tween in PBS. For visualisation of focal adhesions and stress fibres, the cells were stained with FITC conjugated antivinculin antibody and TRITC conjugated Phalloidin and the frequency of stress fibre and focal adhesions were determined as described previously [8].

### Multiplex Reverse Transcription PCR (RT-PCR)

Total cellular RNA was extracted from tumour cell lines as described [8]. Total RNA (5 µg) was reversed transcribed using SuperScript II Reverse transcriptase and oligo-dT. Subsequently, the relative amount of Dlc1 isoform 2 and 3 was quantified in multiplex RT-PCR using primers specific to each isoform. A list of all primers used can be found in Table 4.

**Table 4:** Lists of Primers Used.

List of primers	Forward Primer	Reverse primer	Sequencing Primer
**KRas2 mutation analysis**			
KRas2 F:	GCCATTTCGGACCCGGAGCGA		
KRas2-R-biotin:		CCTACCAGGACCATAGGCACATC	
KRas2 sequencing primer	CTGCTGAAAATGACTGAG		
**Methylation analysis:**			
Isoform 2	Biotin-GTAAGAAAAGGTAGAGGGGAAATTGAGTA	CTCCTTCCCCCTTTCCTAAAATAT	TTTTCCCAAAATCTACACT
Isoform 3	TAGGAGGTTAGTATGGGTTGTA	Biotin-AACCTAATACTAACATAAAACAATATC	AAATTGTTTTTTTGATTTTAA
**Deletion mapping**			
D8Mit293	CGTCATTCTTATAAATCTACCCCC	TTTGCCTGTTTATTGGTCAGG	
**D8Mit296**	GGCAACAAAATCAAAAGCGT	ATTGTATGGAGCACTACTATTTGGG	
**D8Mit97**	ATCTTTTAACTTAGGTGGAGAAAAACC	CTGTGCAAAGTTGCTAAAACAC	
**D8Mit225**	CCCTCTTCTTCCCTTCCACT	TTTGTTGTTGCTTGCTTTGG	
**D8Mit174**	CTCTTTCATGCTCTCTTCTATTGC	ATATACCCAATGCATAAACATATATGC	
**Dlc1**	ACCTGCATGCTGATCTTCTCG	GCTACACACAATCCCTCTGCC	
**Multiplex PCR:**			
Isoform 3 F	CATCAGAGACTCCACCGCCAG		
Isoform 2 F	CTTCTGGCAGCCTCGACGTTC		
Exon 2 R		TGCATACTGGGGGAAACCAGTC	

### Western Blotting and Immunohistochemistry

The T lymphoma as well as the thymic epithelial cells were grown on 150 mm plates/flask until confluent and the total cellular protein was extracted as described [8]. The western blots were hybridized with Dlc1 antibody (Sc32931, Santa Cruz Biotechnology, CA, USA) and p21^waf1^ antibody (Acris Antibodies Gbmh, AP06263PU-N) and visualized. The lysophosphatidic acid (LPA) induced Rho activity in different lymphoma and thymic epithelial cell lines were analysed using the active Rho-GTP pull down assay [8]. The western blots were scanned using a Storm 840 PhosphorImager scanner and quantified by densitometry using ImageQuant software (version 1.2; both from Molecular Dynamics, Inc, CA, USA).

The tumour samples were fixed in 10% neutral buffered formalin for 48 h. The samples were then embedded in paraffin and cut into 6 µm sections and stained with haematoxylin and eosin (H&E) to visualize general morphology. For lung metastasis analysis, five step sections of the entire lung separated by 50 µm were examined and metastases were counted. Lung metastasis was identified by microscopic analysis of H&E sections of 6 µm. For immunohistochemistry, the sections were deparaffinised and incubated in 0.3% hydrogen peroxide in methanol for 10 min at room temperature to block endogenous peroxidase activity. After incubation in normal blocking serum for 30 min, sections were incubated with primary antibody at 37°C overnight. (CD3 antibody ab5690 Abcam, Cambridge, MA, USA); Troma-1, specific for cytokeratin 8 [53] (obtained from Developmental Studies Hybridoma Bank, University of Iowa, Iowa City, Iowa, USA); cytokeratin 5 (H-40, sc-66856 Santa Cruz Biotechnology, Santa Cruz, CA, USA) followed by washing three times in phosphate-buffered saline supplemented with Triton X-100 (PBST) for 3–5 min, and finally incubated with secondary antibody (Dako EnVision+ peroxidise Rabbit; Code:K4002; Carpentaria, CA) at room temperature for 30 min. The bound antibodies were detected using DakoCytomation Envision+ system-HRP labelled polymer (Code: K4002). The sections were washed, saturated with 3, 30-diaminobenzidine tetra hydrochloride for 3 min, and then counterstained with haematoxylin.

### Microdissection and DNA Extraction

The normal cells were removed from tumours by micro-dissection and the samples containing >80% tumour cells were taken for DNA extraction [54]. DNA was extracted from the micro-dissected tissue sections, corresponding normal tissue and cultured cells by Proteinase K digestion followed by phenol-chloroform extraction (Sambrook et al. 1989).

### Loss of Heterozygosity Analysis

We performed microsatellite based deletion mapping to determine if there was loss of heterozygosity at the Dlc1 locus. Six microsatellite markers, namely D8Mit293, D8Mit225, Dlc1 [55], and D8Mit226, D8Mit97, and D8Mit194 were used, which spans 1.9 Mb chromosomal region around Dlc1 locus (based on Build 37 assembly by NCBI). Polymerase chain reaction (PCR) was performed using mouse genomic DNA from the tumour and the corresponding normal tissue. The reactions (25 µl) employed *Taq* DNA polymerase and buffer from Takara (Madison, WI, USA), 50 ng of mouse genomic DNA, and primers at a final concentration of 0.4 µM each. One of the paired primers in the reaction mixture was end labelled with [γ^32^P] ATP using T4-Polynucleotide Kinase. The labelled PCR products were electrophoresed in 7% polyacrylamide gel containing 8 M urea and the gel was exposed to phosphoimager and scanned using a Storm 840 PhosphorImager scanner (Molecular Dynamics, Inc, Sunnyvale, CA, USA). The intensity of the allele specific band was quantified using ImageQuant software (version 1.2; from Molecular Dynamics, Inc). The allelic loss was recorded if there was a complete absence of one allele or if the relative band intensity of one allele was reduced at least 50% in the tumour in comparison to the homologous allele in the corresponding normal DNA.

### DNA Methylation Study of Dlc1 Promoter Region

The genomic DNA from microdissected primary tumours as well as from the lung metastases and tumour derived cell lines were bisulfite treated and then the target region was PCR amplified using biotinylated primers and subsequently sequenced using pyrosequencing technique [8].

### Transendothelial Migration and Invadopodia (in Vitro Filipodia) Formation Assay of the T Lymphoma Cells

Trans-endothelial migration of T-cells was measured as described [56]. Tissue culture wells (8 µm pores, Becton Dickinson Labware, Franklin Lakes, NJ USA) coated with 2 mg/ml fibronectin were seeded with bEnd.3 cells and grown to confluence, which were then activated overnight with 10 ng/ml TNF-α before adding the cells to the upper chamber. Lymphocytes (1×10^6^ cells) were seeded on the upper compartment on top of the endothelial monolayer in 100 µl. After 16 hours of treatment, the tissue culture wells were removed and the lower compartment cells were collected to determine the number of transmigrated lymphocytes. To assess the formation of leading edge filipodia, the T lymphoma cells were labelled with cell tracker green and added to confluent monolayers of tumour necrosis factor α (TNF-α) (cat 300-01A PeproTech, Rocky Hill, NY, USA) activated bEnd.3 cells grown on glass cover slips that had been coated with 2 µg/ml fibronectin. After, 16 hours of incubation, non adherent cells to the cover slips were removed by gentle washing the adherent cells were fixed with 4% paraformaldehyde and stained with DAPI and TRITC-phalloidin. Z stack images were captured using Axiovision 4.1 Zeiss microscope and analyzed.

### Treatment of Cell Culture with 5 Azacytidine (5-AzaC)

The TL 1–4 and TEC 1–3 cells were seeded at 6×10^6^ cells per 100 mm dish. Twenty-four hours later, freshly prepared 5-AzaC (Sigma Chemical Co., St. Louis,Mo.) was added to the cultures at a final concentration of 3 µM. The drug was removed after 24 Hrs and cells were subcultured for 5 subsequent passages with additional incubation in 5 AzaC at the 4^th^ passage. At passage 5, the cells were harvested and total cellular RNA was extracted which was followed by cDNA preparation and subsequent the Dlc1 splice variants were studied by multiplex PCR as mentioned previously (Figure-4C/iii).

Total cellular protein lysates harvested from 5-AzaC treated cells were also incubated with Calf intestinal alkaline phosphatase (CIP; GE Healthcare, Little Chalfont, Buckinghamshire, United Kingdom) at 37°C for 1 h, and resolved in SDS page and immunoblotted with anti Dlc1 antibodies (Sc32931, Santa Cruz Biotechnology, CA, USA).

### Statistical Analysis

The one-way analysis of variance (ANOVA) test was used to compare different cell lines with respect to the mean of Dlc1 protein expression, p21^waf1^ protein expression, focal adhesion frequency, stress fibre frequency and the cell migration rate. Two-way ANOVA was used to assess the effect of LPA on active Rho induction in three different lymphoma and TEC cell lines compared to controls. All the statistical analysis was performed using Sigma Stat 7 for Windows software, version 2.03, 1997, SPSS Inc, IL, USA. Statistical significance was defined as P<0.05.

## Supporting Information

Figure S1 **Immunopreciptation of Dlc1 protein and mass spectrometry:** A: Silver stained polyacrylamide gel showing the immunopreciptated proteins. The primary antibody used was polyclonal anti mouse Dlc1 (Santa Cruiz) which was cross-linked to Dyanbeads Protein G (100.09D, Invitrogen) to pull down the antibody bound protein, ID immunodepleted, IP immunoprecipitated. B: Western blot showing the immunoprecipitated Dlc1 proteins in different cell lysates. C: The eluted immunoprecipitate was subjected to trypsin digestion followed by tandem mass spectrometry analysis using AB SCIEX TripleTOF™ 5600 System (Applied Biosystems/MDS Sciex, Foster City, CA), and identified 7 Peptide sequences representing Dlc1 are underlined. D: Summary of Dlc1 peptides detected from by mass spectrometry.
(TIF)
