# Supplementary figures and images for: The Role of Dlc1 Isoform 2 in K-Ras2G12D Induced Thymic Cancer
Source: PLoS One. 2012 Jul 5;7(7):e40302. doi: 10.1371/journal.pone.0040302 (PMC3390377; doi:10.1371/journal.pone.0040302)

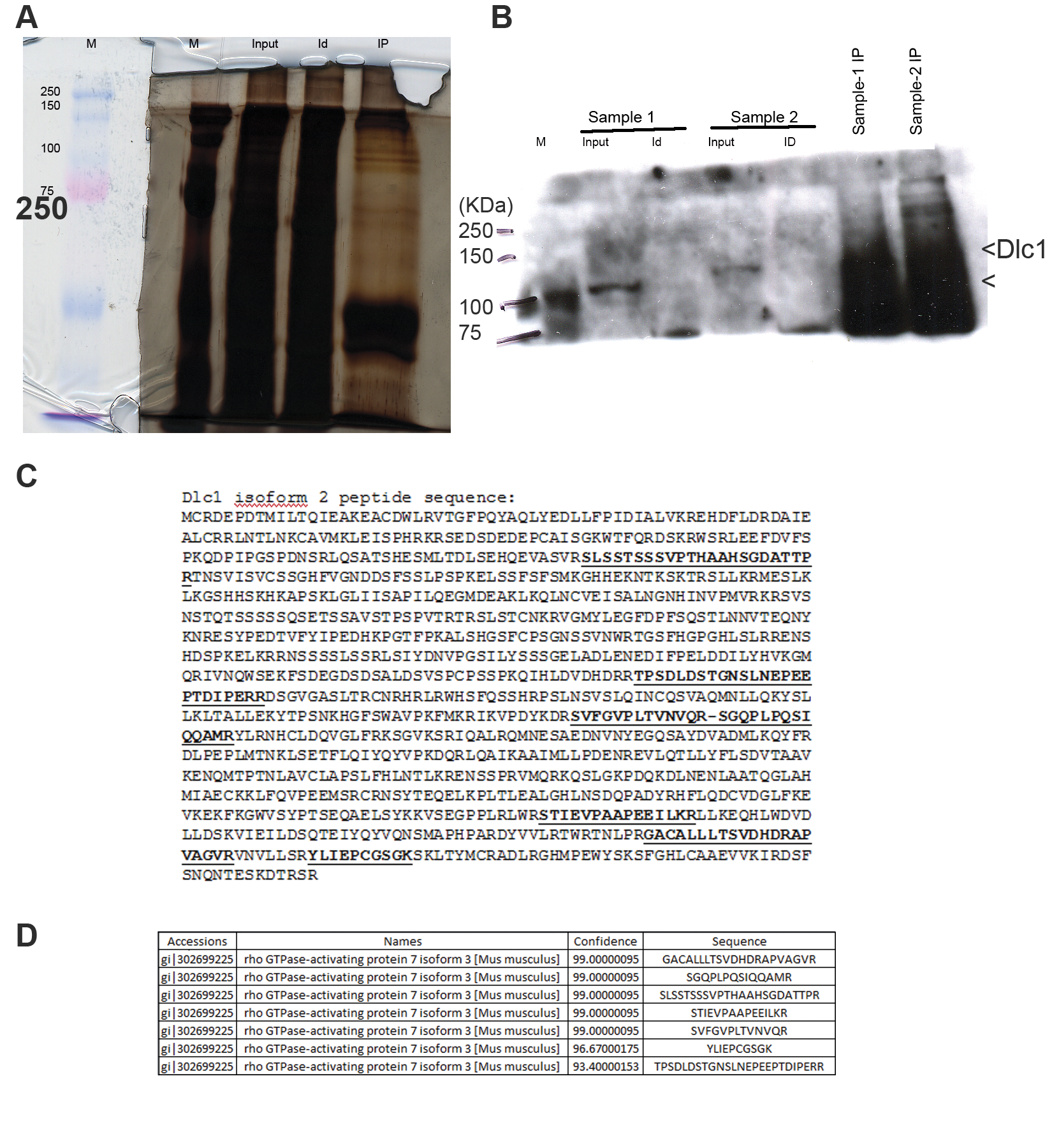

Supplement: Figure S1 — Immunopreciptation of Dlc1 protein and mass spectrometry: A: Silver stained polyacrylamide gel showing the immunopreciptated proteins. The primary antibody used was polyclonal anti mouse Dlc1 (Santa Cruiz) which was cross-linked to Dyanbeads Protein G (100.09D, Invitrogen) to pull down the antibody bound protein, ID immunodepleted, IP immunoprecipitated. B: Western blot showing the immunoprecipitated Dlc1 proteins in different cell lysates. C: The eluted immunoprecipitate was subjected to trypsin digestion followed by tandem mass spectrometry analysis using AB SCIEX TripleTOF™ 5600 System (Applied Biosystems/MDS Sciex, Foster City, CA), and identified 7 Peptide sequences representing Dlc1 are underlined. D: Summary of Dlc1 peptides detected from by mass spectrometry. (TIF) [file pone.0040302.s001.tif]
